# Estimating Genotypic Diversity of Streptococcus mutans Isolated From Caries-Active and Caries-Free Individuals Among Indian Population

**DOI:** 10.7759/cureus.22436

**Published:** 2022-02-21

**Authors:** Saravanan Poorni, MS Nivedhitha, Manali R Srinivasan, Arthi Balasubramaniam

**Affiliations:** 1 Conservative Dentistry and Endodontics, Saveetha Dental College, Chennai, IND; 2 Conservative Dentistry and Endodontics, Sri Venkateshwara Dental College, Chennai, IND; 3 Community Dentistry, Saveetha Dental College, Chennai, IND

**Keywords:** streptococcus mutans, genotypic diversity, dental caries, colony forming units, caries active individuals

## Abstract

Purpose

To identify the genetic characterization of S mutans strains isolated from the caries-free and caries-active population using arbitrarily primed -polymerase chain reaction (AP-PCR).

Materials and methods

Thirty-five subjects with a decayed missing and filled teeth (DMFT) score of 0 were allocated to the caries-free group and 35 subjects with a DMFT score greater than four were allocated to the caries-active group and salivary samples were collected. The samples were processed and the colony forming units (CFU) of S mutans were determined for all the samples. About three colonies resembling S mutans from each subject were subjected to deoxyribonucleic acid (DNA) isolation, a purification process was performed, and DNA was analyzed by AP-PCR.

Results

Among the 210 isolates from 35 caries-active and 35 caries-free subjects that were analyzed by AP-PCR, 41.9% had a single genotype, 25.8% had two genotypes, 19.4% had three genotypes, 9.7% had four genotypes and 3.2% had more than four genotypes among the caries-free group. Among the caries-active group, 54.3% had a single genotype, 25.7% had two genotypes, 14.3% had three genotypes and 5.7% had four genotypes.

Conclusion

The study concluded that different genotypic pattern was present in caries-free and caries-active subjects which indicate the occurrence of genetic polymorphism among the S mutans strains. There was no particular genotype of S mutans that was uniquely present in subjects in caries-active and caries-free individuals.

## Introduction

Dental caries is considered a chronic, multifactorial, localized, post-eruptive transmissible infectious disease that leads to the destruction of dental hard tissue [1]. The two microbial species which are predominantly associated with dental caries are Streptococcus mutans (S mutans) and Streptococcus sobrinus (S sobrinus) which are frequently isolated from dental plaque. These species are found to produce large amounts of acids and extracellular polysaccharides and this, in turn, promotes dental caries [2,3]. Streptococcus mutans in addition to exhibiting the property of acidogenicity and acidurity also consume sucrose to produce insoluble glucans and this plays a vital role in the caries development [4-6].

Bacterial typing is done to determine the association of certain strains of bacteria with specific diseases and also characterize the heterogeneity of various infections [7]. Typing of S mutans may be useful to study the characteristics of S mutans genotypes, in terms of the concepts of diversity and to determine the number of different S mutans genotypes found in an individual. The commonality is generally a term used to specify the number of Streptococcus mutans genotypes that are shared among individuals in a population. Stability is nothing but the persistence of S mutans genotypes over time in a particular individual. Bowden described the necessity to understand the patterns of clonality among S mutans in the caries-free subjects [7].

Literature search showed that the genetic diversity of S mutans have been extensively researched and found that S mutans have significant genetic diversity at the clonal level even within the same mouth. Napimoga et al. [8] in their study demonstrated that more genotypes were found in caries-active subjects than those caries-free subjects. On the other hand, Kreulen et al. and other researchers in their study showed a negative correlation between caries activity and the genotypic diversity [9-12]. The possible association between the biodiversity of S mutans and caries activity among the Indian population deserves attention, as there is paucity in the literature.

Among the several molecular methods that enabled the study of oral streptococci, restriction endonuclease analysis and ribotyping have revealed genetic heterogeneity within S mutans. Arbitrarily primed polymerized chain reaction technique (AP-PCR) has been an extensively applied method for genotyping the bacterial species that includes even the oral pathogens. Genotyping by AP-PCR technique is currently being used to demonstrate different DNA profiles in clinical isolates of S mutans [13-16]. Since there are no studies in the literature that has evaluated the genotypic diversity of S mutans in the Indian population, the present study was undertaken. The aim of the present study was to identify the genetic characterization of S mutans strains isolated from caries-free and caries-active populations using AP-PCR.

## Materials and methods

Study population

This cross-sectional study commenced after obtaining the Ethical approval from the Institution’s Ethics Committee of the author’s institution. Subjects within the age group of 18-28 years were included in the study. This age group was selected because of their high risk for caries activity. Subjects failing to give consent for the study, requiring emergency dental management, those with developmental disorders of teeth, under antibiotic use, and those under medication that modifies salivary secretion were excluded from the study. Subjects with periodontal disease and general/systemic diseases were also excluded from the study. A hundred and seven healthy volunteers who fulfilled the inclusion and exclusion criteria of the study were randomly selected from the population and screened. Subjects were elaborated regarding the procedures and written informed consent was obtained from all the subjects before initiating the study.

Grouping

The subjects were assessed for the decayed missing and filled teeth (DMFT) scores and based on the DMFT scores the subjects were grouped into two - caries-free and caries-active individuals. Each group consisted of 35 subjects. Results obtained from the pilot study were used to calculate the sample size by keeping 80% power and 95% confidence interval. Thirty-five Subjects with a DMFT score of 0 were allocated to the caries-free group and 35 subjects with a DMFT score greater than four were allocated to the caries-active group. The rest 27 subjects were excluded from the study.

Sample collection

Subjects were previously instructed not to eat anything in the morning and not to brush their teeth before sampling. Salivary samples were collected between 7 am and 9 am on different days. Before the sample collection, subjects were asked to relax for about 5 min. They were instructed to swallow the saliva present in their mouth before initiating the sample collection. The subjects were told to spit the saliva in a sterile graduated container. One milliliter of unstimulated whole saliva was collected from each subject and stored on ice until it was transported to the bacteriological laboratory. Transportation of the samples to the laboratory was done immediately and they were processed within 3 hrs from the time of collection.

Sample processing

Saliva was transferred to a sterilized centrifuge tube and agitated in a vortex test tube mixer for 30 s and serially diluted. In order to detect S mutans, 5 µl of undiluted samples were taken. 10-1 to 10-3 dilution samples were cultured on Mitis salivarius agar plates with 0.2 units ml-1 of bacitracin (MSB agar). The plates were incubated in an anaerobic condition at 37°C for 48 hrs in a 10% CO2 atmosphere.

Anaerobic condition

When citric acid and sodium bicarbonate combine with each other and with water CO2 is produced this CO2 is used in the cultivation of S mutans in anaerobic conditions inside a tightly sealed box. The culture plates are kept inside the box. This is followed by a beaker containing a mixture of sodium bicarbonate, citric acid, and water. Then the CO2 incubator is tightly sealed using silicone. The incubator is maintained below 45ºC.

Streptococcus mutans colony count

Following incubation, when samples exhibited growth on the Mitis salivarius bacitracin (MSB) agar plates, the microbial colonies on each plate were counted using a colony counter. Whenever possible, plates containing 30 - 300 colonies were selected for enumeration. The number of colony forming units per milliliter (CFU/ml) expressed was calculated by multiplying the number of colony forming units (CFU) with the original ml of sample used for dilution.

Streptococcus mutans isolation

Ten to fifteen isolates per sample representing all morphological types (those with rough appearance, those that were adherent to the agar, and those with glistening drops of liquid on or around the colony) were selected. When there were differences in colony morphology, efforts were taken to include each presumptive colony type. Each and every colony was transferred to 3 ml of brain heart infusion broth (BHI broth) and incubated for 24-hour in an atmosphere of 10% CO2. Biochemical tests were used to confirm the purity and identity of the cultures and stored at -80°C in 20% glycerol until further use.

DNA extraction

The procedure for DNA extraction was based on the study by Ravikumar et al. [17]. From each subject, about three colonies that resembled S mutans were transferred to brain heart infusion (BHI) broth (Difco, USA). It was incubated in an anaerobic jar for 48 hrs at 37◦C. Genomic DNA preparation, isolation, and purification of DNA from 210 isolates were done. Bacterial culture was randomly selected for each subject and 1.5 ml was transferred to a microcentrifuge tube and was spun at 10,000 rpm 4°C for 2 min. The supernatant solution was transferred. The pellet was then resuspended in about 467 ml of the Tris ethylenediamine tetraaceticacid (EDTA). Thirty milliliters of 10% Sodium lauryl sulfate (SDS) and 3 ml of Proteinase K, 20 mg/ml was then added to the sample. The sample was incubated for about an hour at 37°C. Phenol: chloroform was added in equal volumes in the ratio 24:1 and mixed gently. To achieve this, invert the tubes until the phase gets completely mixed. After this, the tubes were then spun at 12,000 rpm for about 10 min at 4°C. The cellular RNA was then removed and the upper aqueous layer was transferred to a fresh tube. Following this, chloroform was added in equal volume. Again the samples were mixed in a similar method by inverting the tubes and spinning at 12,000 rpm for about 10 min at 4°C. The aqueous phase that was present in the upper part was transferred to a new tube and about 1/10th volume of 3M sodium acetate was then added to the solution. 95% ice-cold ethanol in double the volume was then added and mixed as previously by inversion while the DNA was precipitated. The tube was then spun again for about 10 min at 12,000 rpm at 4°C. The supernatant solution was then discarded. This was followed by washing the pellet with 0.2 ml of 70% ethanol and the tube was spun again as before. This 70% ethanol was then discarded and the pellet was air-dried. The quality of the DNA was then confirmed by agarose gel electrophoresis by suspending in Tris EDTA TE buffer and run on 0.8% agarose gel followed by viewing under Ultraviolet light. A digital camera was used to record the gel pictures of extracted DNA. The ratio of absorbance at 260 and 280 nm Ultraviolet light was used to calculate the purification of DNA and the value was found to be 1.8. The cultures obtained from all the subjects were subjected to the same procedure.

PCR amplification and visualization of amplified products

The extracted DNA from the isolates of S mutans was proceeded for PCR amplification. PCR with species-specific primers (gtfB and gbpB) were conducted for the identification of S mutans. (5’- ACTACACTTTCGGGTGGCTTGG-3’ and 5’- CAGTATAAGCGCCAGTTTCATC) are the primers that are specific for gtfB, encoding glucosyltransferase B. (5’- CAACAGAAGCACAACCATCA-3’ and 5’- TGTCCACCATTACCCCAGT-3’) are primers that are specific to gbpB, encoding glucan binding protein B. Though gtfB primers amplify S mutans gtfB gene, yet previous studies revealed that gtfB primers generate some cross-amplification with several clinical isolates of S sobrinus. This was defined as S sobrinus species by sequencing of 16SrRNA. In order to overcome this hindrance, the strains were also tested with gbpB primers. These primers yield amplicons of predicted size in all S mutans genotypes and they do not amplify S sobrinus sequences.

AP-PCR analysis

Arbitrarily primed polymerase chain reaction (AP-PCR) with OPA 13 arbitrary primer were then used to genotype the strains. The isolates were then submitted to genotyping protocols. PCR amplification was performed in a reaction mixture consisting of PCR beads that contained an enzyme and the required reagents, 25 p mol of each primer, and 20-50 ng of the template DNA solution in a thermal cycler. Positive and negative controls were included in each PCR set and for all sample processing. S mutans JCM5175T was used as control. The reaction mixture was denatured at 95ºC for 2 min followed by a series of amplification: denaturation at 95ºC for 1 min, annealing at 36ºC for 2 min, and extension at 72ºC for 2 min. The series was repeated for 26 cycles. The final cycle comprised 94ºC for 1 min, 55ºC for 1 min, and 72ºC for 5 min. After amplification, the PCR products were analyzed by electrophoresis on an agarose 1.2% gel. The newly visualized DNA fragments were visualized under ultraviolet light at 302 nm after staining with ethidium bromide. The size of the PCR products was estimated from the electrophoretic migration of products relative to a 100-bp ladder. Isolates were considered as having the same genotypic identity when presented with identical AP-PCR product size profiles. Any repeatable difference regarding the strong bands was considered discriminatory. The genotypes found were analyzed descriptively.

Data on CFU was recorded on Microsoft Excel and was subjected to statistical analysis for evaluating the difference between caries-active and caries-free groups. The difference in the mean CFU between the two groups was statistically analyzed using Independent Student ‘t-test. The differences between the genotypic diversity were evaluated by Fisher Exact test. Statistical significance was considered to be at α < 0.05. SPSS software version 23 was used for the data analysis.

## Results

Among the 70 subjects enrolled for the study, 40 were males and 30 were females. In both caries-active and caries-free groups, 57% of the subjects were males and 43% were females. The age of the subjects enrolled in both the groups ranged from 18 yrs to 28 yrs. The overall mean age of the subjects was 23.4 yrs. The mean age of the subjects belonging to the caries-active group was 23.5 yrs while that of the subjects belonging to the caries-free group was 23.3 yrs. In all, 70 samples were analyzed from 35 caries-free and 35 caries-active subjects. All the subjects belonging to the caries-active group harbored S mutans in the saliva while S mutans were not detected in one subject belonging to the caries-free group.

The CFU value of S mutans was found to be higher in caries-active subjects than in caries-free subjects. The difference in the mean CFU between the two groups was found to be statistically significant as shown in Table 1.

**Table 1:** Table showing the mean S mutans colony count (CFU/ml) in 105 in caries-active and caries-free groups * Independent sample t-test with p-value < 0.005 - level of significance

Groups	N	Mean CFU/ml in 10^5^	Std. Deviation	p Value
Caries-active group (C)	35	3.5	0.94	0.000^*^
Caries-free group (NC)	35	1.5	0.69	

A total of 10 isolates of the caries-active group and 105 isolates of the caries-free group were analyzed by AP-PCR. Genomic DNA was extracted and purified from the cell pellet and stored at -20ºC. Integrities of the genomic DNA samples were checked in samples electrophoretically resolved in 1% agarose gel and stained with ethidium bromide (5 μg/mL). Isolates were confirmed for species identity in PCR reactions with primers specific for gtfB, enconding glucosyltransferase B (5’- ACTACACTTTCGGGTGGCTTGG-3’and5’- CAGTATAAGCGCCAGTTTCATC and specific to gbpB, enconding glucan-binding protein B (5’- CAACAGAAGCACAACCATCA-3’ and 5’- TGTCCACCATTACCCCAGT-3’). Isolates were identified as S mutans in the PCR reactions with specific primers.

**Figure 1:**
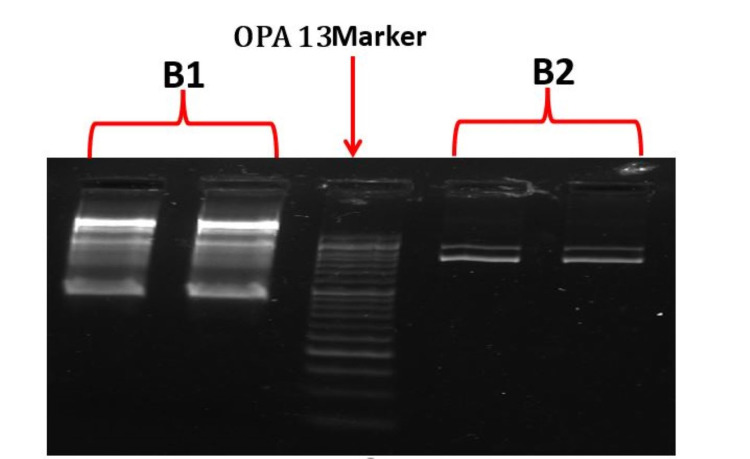
Representative AP-PCR profiles (Amplitypes) identified among S mutans strains

Among the 210 isolates from caries-active and caries-free subjects that were analyzed by AP-PCR, the S mutans detected by AP-PCR were belonging to 135 different genotypes. We detected 65 genotypes of S mutans in caries-free subjects and 60 genotypes in caries-active individuals. Figure 1 shows the AP-PCR patterns performed with OPA-13 which generated a different spectrum of amplicons, indicative of genetic polymorphism.

Table 2 shows the diversity of genotypes among caries-active and caries-free groups. This analysis resulted in the identification of 13 subjects (41.9%) who had a single genotype, eight subjects had two genotypes (25.8%), six subjects (19.4%) had three genotypes, three subjects (9.7%) had four genotypes and one subject (3.2%) had more than four genotypes among the caries-free group. Among the caries-active group 19 subjects (54.3%) had a single genotype, nine subjects (25.7%) had two genotypes, five subjects (14.3%) had three genotypes and two subjects (5.7%) had four genotypes. The results show that more genotypes are present in caries-free subjects than in caries-active subjects. There was no statistically significant difference between the two groups of individuals (p=0.772)

**Table 2:** Table showing the diversity of genotypes among caries-active and caries-free groups 3 Isolates from each of the 35 caries-active subjects and 35 caries-free subjects (N = 70, total 210 isolates)

Number of Genotypes	1	2	3	4	More Than 4
Number of caries-free Subjects	13	8	6	3	1
Number of caries-active Subjects	19	9	5	2	0
Total individuals by genotypes	32	17	11	5	1

## Discussion

The primary microorganisms in the oral cavity are the Mutans streptococci, particularly S mutans. They facilitate the maintenance of normal ecological balance. Hence, they are considered to be the principal etiological agents of dental caries [18]. Literature search showed that S mutans can be isolated from both caries-active and caries-free individuals. Previous studies have also shown a difference in the S mutans genotypes isolated from caries-active and caries-free individuals [10,13]. Since there were no studies in the literature that evaluated this diversity in the Indian population, the present study was undertaken.

Determining the number of S mutans in saliva and plaque has been used to predict individuals’ susceptibility to caries for many years [19]. In our study, it was found that the CFU value of S mutans was higher in caries-active subjects than in caries-free subjects. This difference in the mean CFU between the two groups was found to be statistically significant. Though the number of S mutans differed among caries-active and caries-free individuals, yet the number of S mutans may not be the only determinant for the caries process. The character of the microorganisms may influence this process [20].

Colonization of particular genotypes of S mutans leading to caries activity is a commonly identified character in dental caries [6]. Our study was thus designed to compare the genotypic diversity of S mutans in caries-active and caries-free individuals using AP-PCR. AP-PCR has been used to evaluate the genotypic profile of S mutans in this study. Several comparisons done with other genotyping techniques have assured the validity of the AP-PCR technique in genotypic identification of microorganisms [13,16, 21-23]. 

AP-PCR analysis of the 210 isolates from 105 caries-active and caries-free subjects showed 135 different S mutans genotypes. Among 135 genotypes, 65 genotypes of S mutans were detected in caries-free subjects and 60 genotypes in caries-active subjects with each of them harboring 0 to four genotypes. This demonstrates the genetic variability of S mutans in caries-active and caries-free individuals. Among the caries-free group, 13 subjects (41.9%) had a single genotype, eight subjects had two genotypes (25.8%), six subjects (19.4%) had three genotypes, three subjects (9.7%) had four genotypes and one subject (3.2%) had more than four genotypes among the caries-free group. Among the caries-active group 19 subjects (54.3%) had a single genotype, nine subjects (25.7%) had two genotypes, five subjects (14.3%) had three genotypes and two subjects (5.7%) had four genotypes. These results suggest that there is a difference in the number of S mutans genotypes present in caries-active and caries-free subjects. Both caries-active and caries-free subjects did not demonstrate a single dominant genotype. The results of our study were in concurrence with the results of Jiang et al. [24].

The present study also demonstrated that caries-active individuals showed lesser genetic polymorphisms than those individuals without caries. Our results were similar to the results obtained by Jiang et al. [24] and differed from Napimoga’s study which showed that caries-active individuals harbored more genotypes [8].

Grönroos and Alaluusua [25] suggested that S mutans clones might selectively colonize specific hard-tissue sites. Our study failed to analyze the predominance of specific genotype in different sites in the oral cavity. One limitation of the study is the less sample size in a limited population. In addition to this, the clinical status of the oral cavity was also not analyzed. Further research in larger populations with cultural variability is required to generalize the results of the study.

## Conclusions

Thus, within the limitations of the present study, it can be concluded that different genotypic pattern was present in caries-free and caries-active. This indicates the presence of genetic polymorphism among the Streptococcus mutans strains. There was no particular genotype of S mutans that was uniquely present in subjects in caries-active and caries-free individuals.

## References

[1] Fontana M, Young DA, Wolff MS, Pitts NB, Longbottom C (2010). Defining dental caries for 2010 and beyond. Dent Clin North Am.

[2] Fragkou S, Balasouli C, Tsuzukibashi O, Argyropoulou A, Menexes G, Kotsanos N, Kalfas S (2016). Streptococcus mutans, Streptococcus sobrinus and Candida albicans in oral samples from caries-free and caries-active children. Eur Arch Paediatr Dent.

[3] Bottner A, He RY, Sarbu A, Nainar SM, Dufour D, Gong SG, Lévesque CM (2020). Streptococcus mutans isolated from children with severe-early childhood caries form higher levels of persisters. Arch Oral Biol.

[4] Bedoya-Correa CM, Rincón-Rodríguez RJ, Parada-Sanchez MT (2021). Acidogenic and aciduric properties of Streptococcus mutans serotype c according to its genomic variability. Eur J Oral Sci.

[5] Chu J, Zhang T, He K (2016). Cariogenicity features of Streptococcus mutans in presence of rubusoside. BMC Oral Health.

[6] Bowen WH, Koo H (2011). Biology of Streptococcus mutans-derived glucosyltransferases: role in extracellular matrix formation of cariogenic biofilms. Caries Res.

[7] Cheon K, Moser SA, Wiener HW (2013). Characteristics of Streptococcus mutans genotypes and dental caries in children. Eur J Oral Sci.

[8] Napimoga MH, Kamiya RU, Rosa RT, Rosa EA, Höfling JF, de Oliveira Mattos-Graner R, Gonçalves RB (2004). Genotypic diversity and virulence traits of Streptococcus mutans in caries-free and caries-active individuals. J Med Microbiol.

[9] Kreulen CM, de Soet HJ, Hogeveen R, Veerkamp JS (1997). Streptococcus mutans in children using nursing bottles. ASDC J Dent Child.

[10] Pieralisi FJ, Rodrigues MR, Segura VG, Maciel SM, Ferreira FB, Garcia JE, Poli-Frederico RC (2010). Genotypic diversity of Streptococcus mutans in Caries-Free and Caries-Active Preschool Children. Int J Dent.

[11] Valdez RM, Duque C, Caiaffa KS (2017). Genotypic diversity and phenotypic traits of Streptococcus mutans isolates and their relation to severity of early childhood caries. BMC Oral Health.

[12] Villhauer AL, Lynch DJ, Warren JJ, Dawson DV, Blanchette DR, Drake DR (2017). Genotypic characterization and comparison of Streptococcus mutans in American Indian and Southeast Iowa children. Clin Exp Dent Res.

[13] Gamboa F, Chaves M, Valdivieso C (2010). Genotypic profiles by AP-PCR of streptococcus mutans in caries-active and caries-free preschoolers. Acta Odontol Latinoam.

[14] Gamboa FO, García DA, Plazas LA (2020). Características microbiológicas y moleculares de microorganismos de importancia en caries dental y enfermedad periodontal: aportes de investigación en Colombia (Article in Spanish). Univ Odontol.

[15] Manchanda S, Sardana D, Liu P, Lee GHM, Lo ECM, Yiu CKY (2021). Horizontal transmission of Streptococcus mutans in children and its association with dental caries: a systematic review and meta-analysis. Pediatr Dent.

[16] Alissa V, David L, Taylor P, Deborah D, David D. (2021). Mutans Streptococci and Lactobacilli colonization patterns and genotypic characterization of cariogenic bacterial species in American Indian children. Front Dent Med.

[17] Ravikumar D, Mahesh R, Ningthoujam S, Robindro W, Gayathri R, Vishnu PV (2018). Genotypic characterization of Streptococcus mutans in child-mother pair-A PCR based study. J Oral Biol Craniofac Res.

[18] Colombo APV, do Souto RM, da Silva-Boghossian CM (2015). Microbiology of oral biofilm-dependent diseases: have we made significant progress to understand and treat these diseases?. Curr Oral Health Rep.

[19] Guo L, Shi W (2013). Salivary biomarkers for caries risk assessment. J Calif Dent Assoc.

[20] Krzyściak W, Jurczak A, Kościelniak D, Bystrowska B, Skalniak A (2014). The virulence of Streptococcus mutans and the ability to form biofilms. Eur J Clin Microbiol Infect Dis.

[21] Damé-Teixeira N, Arthur RA, Parolo CC, Maltz M (2014). Genotypic diversity and virulence traits of Streptococcus mutans isolated from carious dentin after partial caries removal and sealing. ScientificWorldJournal.

[22] Tabchoury CP, Sousa MC, Arthur RA, Mattos-Graner RO, Del Bel Cury AA, Cury JA (2008). Evaluation of genotypic diversity of Streptococcus mutans using distinct arbitrary primers. J Appl Oral Sci.

[23] Li Y, Caufield PW (1998). Arbitrarily primed polymerase chain reaction fingerprinting for the genotypic identification of mutans streptococci from humans. Oral Microbiol Immunol.

[24] Jiang Q, Yu M, Min Z, Yi A, Chen D, Zhang Q (2012). AP-PCR detection of Streptococcus mutans and Streptococcus sobrinus in caries-free and caries-active subjects. Mol Cell Biochem.

[25] Grönroos L, Alaluusua S (2000). Site-specific oral colonization of mutans streptococci detected by arbitrarily primed PCR fingerprinting. Caries Res.

